# Understanding and controlling the structure and segregation behaviour of AuRh nanocatalysts

**DOI:** 10.1038/srep35226

**Published:** 2016-10-14

**Authors:** Laurent Piccolo, Z. Y. Li, Ilker Demiroglu, Florian Moyon, Zere Konuspayeva, Gilles Berhault, Pavel Afanasiev, Williams Lefebvre, Jun Yuan, Roy L. Johnston

**Affiliations:** 1 Institut de recherches sur la catalyse et l’environnement de Lyon (IRCELYON), UMR 5256 CNRS & Université Claude Bernard - Lyon 1, 2 avenue Albert Einstein, F-69626 Villeurbanne, France; 2 Nanoscale Physics Research Laboratory, School of Physics and Astronomy, University of Birmingham, Birmingham B15 2TT, United Kingdom; 3 School of Chemistry, University of Birmingham, Birmingham B15 2TT, United Kingdom; 4 Normandie Université, UNIROUEN, INSA Rouen, CNRS, Groupe de Physique des Matériaux, 76000, Rouen, France; 5 Department of Physics, University of York, York, YO10 5DD, United Kingdom

## Abstract

Heterogeneous catalysis, which is widely used in the chemical industry, makes a great use of supported late-transition-metal nanoparticles, and bimetallic catalysts often show superior catalytic performances as compared to their single metal counterparts. In order to optimize catalyst efficiency and discover new active combinations, an atomic-level understanding and control of the catalyst structure is desirable. In this work, the structure of catalytically active AuRh bimetallic nanoparticles prepared by colloidal methods and immobilized on rutile titania nanorods was investigated using aberration-corrected scanning transmission electron microscopy. Depending on the applied post-treatment, different types of segregation behaviours were evidenced, ranging from Rh core – Au shell to Janus *via* Rh ball – Au cup configuration. The stability of these structures was predicted by performing density-functional-theory calculations on unsupported and titania-supported Au-Rh clusters; it can be rationalized from the lower surface and cohesion energies of Au with respect to Rh, and the preferential binding of Rh with the titania support. The bulk-immiscible AuRh/TiO_2_ system can serve as a model to understand similar supported nanoalloy systems and their synergistic behaviour in catalysis.

Heterogeneous catalysis plays a key role in strategic fields such as pollution control, materials and chemicals synthesis, and fuel production. The combination of several metals has long been recognized as an important route for improving the activity, selectivity and/or stability of supported nanocatalysts through various geometric and electronic effects^1^,^2^,^3^,^4^. In recent years, the renewed interest for nanoalloys, *i.e.* well-defined multimetallic nanoparticles, has been driven by great advances in atomic-scale characterization techniques (*e.g.* aberration-corrected transmission electron microscopy, AC-TEM) and computer simulation methods (based *e.g.* on density functional theory, DFT)^5^,^6^,^7^,^8^,^9^,^10^. Together with the development of nanocatalyst preparation methods^11^,^12^,^13^,^14^,^15^,^16^, this progress allows researchers to better model and understand surface reaction processes, thereby enabling a “rational design” of more efficient catalysts.

Although several groups have succeeded in synthesizing solid-solution nanoalloys of bulk-immiscible elements using colloidal methods^11^,^13^,^14^, these structures are generally metastable and cannot resist the thermal treatment needed for removing the stabilizing agents. As the thermodynamic properties of bulk alloys can influence the structure of their nanosized counterparts, some of us have recently compared the well-known bulk-miscible Au-Pd system^7^,^12^,^16^ to the rarely studied bulk-immiscible Au-Rh system^11^,^13^,^17^ in terms of mixing behaviour and reactivity at the nanoscale^9^,^15^,^18^. As seen for 3 nm-sized nanoparticles (NPs) anchored on well-defined rutile titania nanorods, Au and Pd atoms form a solid-solution alloy, whilst Au and Rh atoms segregate into single-phase domains within the NPs^15^. Nevertheless, in both cases the interaction of the active metal (Pd, Rh) with gold and titania inhibits the surface oxidation or sulfidation of the former^15^.

AuRh/TiO_2_ catalysts have proved to be efficient in several reactions including the hydrogenation of tetralin in the presence of H_2_S, during which the bimetallic catalyst showed improved sulfidation resistance^15^, and the hydrodeoxygenation of guaiacol, for which the AuRh catalyst performed more selectively than Au and Rh ones^19^, both carried out in large excess of hydrogen around 300 °C. In this work, with the aim of gaining insights into the structure of the catalytically active phase, the catalyst submitted to thermal treatments in hydrogen has been investigated in details by aberration-corrected scanning transmission electron microscopy (AC-STEM). Moreover, DFT calculations have enabled us to rationalize the striking bimetallic catalyst structures observed experimentally.

## Results and Discussion

### Structural analysis of AuRh nanoparticles

#### Unsupported nanoparticles

**Figure 1:**
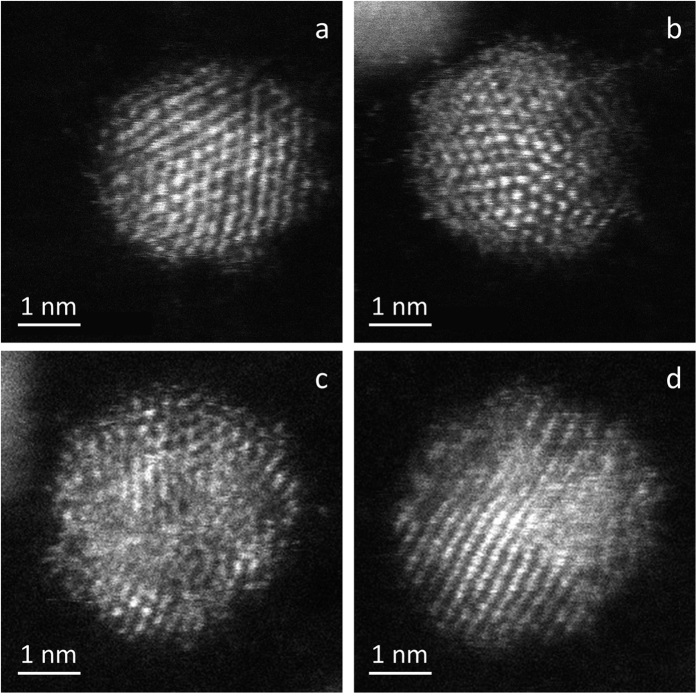
AC-STEM characterization of unsupported nanoparticles. HAADF images of AuRh@PVA colloids (sample 1) deposited on lacey C grid, showing single-crystal (**a**), multi-twinned (**b**), Rh@Au core-shell (**c**), and Janus (**d**) structures.

Au-Rh nanoparticles were synthesized by conventional colloidal chemical co-reduction in water, using chloride salts as metal precursors, polyvinyl alcohol (PVA) as surfactant, and NaBH_4_ as reducing agent, as previously reported^15^ (see Methods section). Figure 1 shows representative AC-STEM images of as-prepared AuRh NPs embedded in PVA (dried AuRh@PVA colloids, sample 1, Au_63_Rh_37_ average composition as determined by elemental analysis), which are round-shaped and *ca.* 3 nm in size. A variety of structures were identified, including alloyed fcc single-crystal, alloyed multiply twinned, and probable Rh@Au core-shell and Au-Rh Janus configurations. The structural inhomogeneity of as-prepared NPs is inherent to the NP synthesis method^12^. It likely arises from the fact that the atom migration is limited by the low (generally room) temperature of the reactant solution.

#### Supported nanoparticles heated to 350 °C

For the preparation of AuRh/TiO_2_ supported catalysts, a powder of single-phase rutile titania nanorods synthesized using a hydrothermal method (see Methods section) was added to the acidified colloidal suspension, which led to the immobilization of PVA-embedded NPs on the titania support. The average size of the bimetallic particles was 3.3 ± 1.0 nm (sample 2). Segregation was observed by STEM in the form of Au or Rh single-phase domains randomly distributed within the nanoparticles, as previously reported^15^.

**Figure 2:**
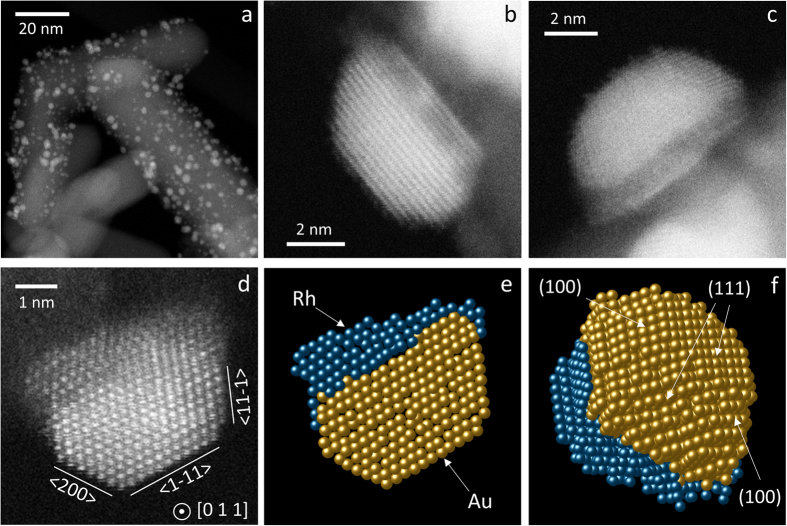
AC-STEM characterization of supported Janus nanoparticles. (**a–d**) HAADF images of AuRh/TiO_2_ (sample 2, Au_63_Rh_37_ average composition) pretreated in H_2_ at 350 °C. Metal particle size: 3.7 ± 1.0 nm. (**e**,**f**) Ball model resulting from the reconstruction of the image in (**d**), viewed along the [011] direction (**e**) and a tilted direction away from the zone axis (**f**).

As previously shown by infrared spectroscopy and TEM, the heating of AuRh/TiO_2_ to 350 °C under H_2_ flow leads to the complete removal of the PVA surfactant from the NP surface without significant change of the mean particle size^15^. However, a drastic change in the morphology and chemical structure of the NPs occurs, as shown by the AC-STEM high-angle annular dark field (HAADF) images of Fig. 2a–d and Supplementary Fig. S1. The NPs are faceted and frequently adopt a Janus-type structure with Rh mostly located at the interface between TiO_2_ and Au, as suggested by the Z-contrast of the images in Fig. 2b–d (Au atoms appear brighter than Rh atoms)^20^.

A statistical analysis^21^,^22^,^23^ was performed on the high-resolution image of the particle shown in Fig. 2d. After determining the number of atoms for each atomic column, a simple back-projection was carried out (see details of the procedure in Supplementary Information, including Supplementary Figs S2–S4). The bright part of the particle in Fig. 2d is well modelled by an fcc truncated octahedron (the most stable structure for Au particles of a few nanometres)^24^,^25^ of *ca*. 1700 Au atoms with a (111) facet parallel to the titania surface (Fig. 2e,f). With respect to regular truncated octahedron, it is further truncated along a (111) plane by a thick layer of Rh (containing *ca*. 700 atoms from the atom-counting analysis). Thus, the hypothesis of a faceted Janus nanoparticle with Rh located at the interface between Au and TiO_2_ gives satisfactory results, with a relatively smooth transition between the surfaces of the Rh and Au parts, *i.e.* roughly equal numbers of Rh and Au atoms along the e-beam direction.

#### Supported nanoparticles heated to 700 °C

**Figure 3:**
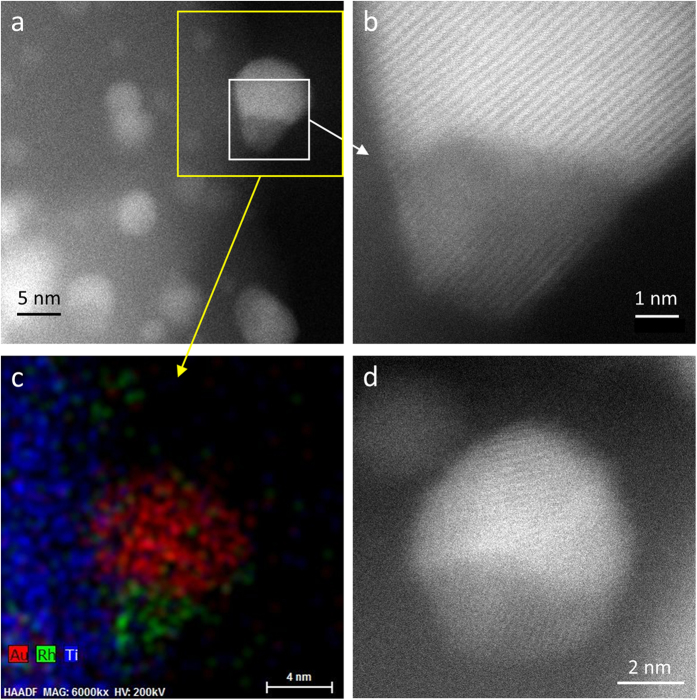
AC-STEM characterization of supported ball-cup nanoparticles. (**a**) HAADF image of AuRh/TiO_2_ (sample 3, Au_55_Rh_45_ average composition) heated in H_2_ flow to 700 °C. (**b**) HAADF image of the region corresponding to the white square in (**a**). (**c**) EDX map of the region corresponding to the yellow square in (**a**). (**d**) HAADF image of an individual particle.

To assess the thermal stability of the catalyst structure above 350 °C, AuRh/TiO_2_ was submitted to constant heating up to 700 °C in H_2_ flow. After this treatment, in addition to pure Au NPs and smaller Rh NPs, the sample presents *ca*. 10 nm-sized bimetallic particles having a segregated structure with Rh or both metals connected to TiO_2_, and the Au side covering partially the Rh side. This is shown by the representative AC-STEM images and EDX maps of Fig. 3 and Supplementary Figs S5–S7. This roundish structure is referred to as “ball-cup”^26^ and noted Rh_ball_Au_cup_ later on. Unlike standard Janus particles, which have a planar (Au-Rh) interface, ball-cup ones have a curved interface with one metal (Rh) partly embedded in the other (Au). The large size of the NPs suggests that these particles may result from the thermally-induced coalescence of smaller ones.

### DFT modelling of free and supported clusters

In order to rationalize the STEM results showing metal segregation in unsupported and supported Au-Rh nanoparticles, a theoretical study was undertaken using DFT computer simulations (DFT-PAW-PBE method, VASP code^27^,^28^,^29^,^30^, see Methods section for details).

#### Unsupported clusters

**Figure 4:**
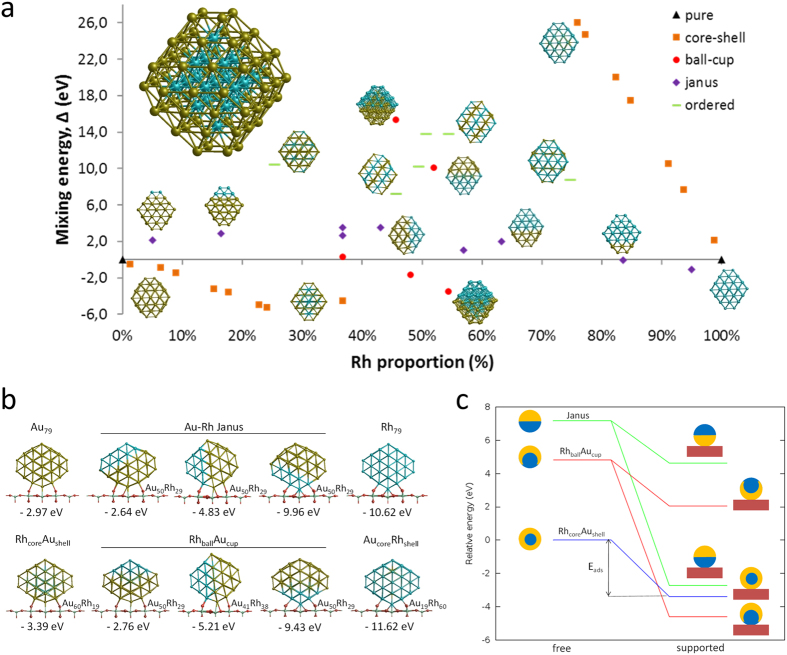
Results of DFT calculations. (**a**) Mixing energy versus atomic composition for 79-atom Au-Rh nanoalloy TO clusters and monometallic counterparts. The most stable cluster (Rh_19_@Au_60_) is enlarged in insert. (**b**) Structure and corresponding adsorption energy for clusters of selected compositions adsorbed on TiO_2_(110). Blue, yellow, cyan, and red spheres represent Rh, Au, Ti, and O atoms, respectively. Only one layer of the TiO_2_ slab is shown for simplicity. (**c**) Schematic view of the energetics of free and supported Au_50_Rh_29_ clusters.

79-atom clusters of truncated octahedral (TO) shape were considered as models for investigating the equilibrium mixing behaviour of Au-Rh nanoparticles. Several nanoalloy models were constructed, covering different compositions and morphologies, such as mixed (ordered alloy), core-shell, Janus and ball-cup particles. These general structural types were then extended to larger TO clusters (up to 260 atoms). The stabilities of various chemical configurations following local geometry optimization are compared in Fig. 4a (the mixing energy^31^ refers to the energy variation due to alloying with respect to the pure clusters, see also Table 1 for selected compositions). As a result, Rh_core_Au_shell_ and Au_core_Rh_shell_ are the most and least stable configurations, respectively. These findings are consistent with the higher cohesive and surface energies of Rh as compared to Au in the bulk state^9^,^15^. Figure 4a shows that the mixing energies of the intermediate structures between the core-shell and the corresponding pure metal particles lie on a straight line for both Rh_core_Au_shell_ and Au_core_Rh_shell_. The second most stable structure is Rh_ball_Au_cup_, the extreme end of this type being the Rh core covered with a half Rh and half Au shell. The Janus-type structures lie slightly higher in energy than the Rh_ball_Au_cup_ structures. The inverse Au_ball_Rh_cup_ type, in which the core is Au-rich, is found to be less stable than the Janus type. When the size of the particle increases (up to 260 atoms), the same stability order is preserved^32^.

#### Supported clusters

Then, Au, Rh and Au-Rh clusters were placed and locally relaxed on the rutile TiO_2_(110) surface, which is a model for the experimentally used TiO_2_ nanorods (that exhibit 80–90% (110) facets)^15^. The clusters were placed between bridging O rows of TiO_2_(110), which is the adsorption position maximizing metal-support interaction. Figure 4b reports the adsorption energies (energy gain due to metal-support interaction) of pure metal clusters and core-shell, Janus and ball-cup nanoalloys. The adsorption strength is higher for the Rh cluster than for the Au cluster one because the Rh-O interaction is stronger than the Au-O one^6^. Consistently, for bimetallic clusters, the adsorption energy is mainly determined by the type of metal atom in contact with the surface. By comparing the mixing energy of the free cluster (Δ) with its supported counterpart (Δ′) one can evaluate the effect of the support on the clusters, *i.e.* determine whether a particular mixing type is stabilized or destabilized. For Janus and Rh_ball_Au_cup_ structures, the mixing energy becomes negative when the clusters are adsorbed on the surface through Rh facets, while it becomes more positive for Au facets (see Fig. 4c and Table 1). Since there is no possibility for Rh-titania contact, Rh_core_Au_shell_ is destabilized on the surface. To determine whether the destabilization of Rh_core_Au_shell_ and the stabilization of Janus and Rh_ball_Au_cup_ through Rh-surface interactions are sufficient to cause a crossover in stability, we constructed Janus, Rh_ball_Au_cup_ and Rh_core_Au_shell_ clusters with the same composition (Au_50_Rh_29_, *i.e.* close to experimental sample composition) and compared their total energies on the surface (Table 1). For this particular composition, Rh_ball_Au_cup_ becomes the lowest total energy structure, surpassing Rh_core_Au_shell_ (see also Fig. 4c). For the Janus structure, although the stabilization is not enough for crossover, the energy gap to Rh_core_Au_shell_ decreases from 7 eV to less than 1 eV upon adsorption.

In summary, while the Rh@Au core-shell structure is by far the most stable in the unsupported state according to DFT calculations, in the supported state this configuration competes with anisotropic segregated structures. Remarkably, the Janus (350 °C annealing in H_2_) and Rh_ball_Au_cup_ (700 °C) configurations experimentally observed for the bimetallic NPs are also predicted for small clusters at equilibrium. This suggests that these structures are close to equilibrium and the energetics is likely controlled by the positive Au-Rh mixing enthalpy and the difference in surface/interface energies rather than by the large lattice mismatch between Au and Rh^15^. Indeed, small clusters can easily accommodate the (small) strain whereas larger nanoparticles relax it through interfacial defects or non-epitaxial relationships between Au and Rh regions, as seen in Fig. 2d for the Janus structure and Fig. 3b for the ball-cup one. Consistent with our direct observation of Au/Rh/TiO_2_ stacking after low-temperature annealing, Han *et al*. suggested from DFT calculations on Au-Ir/TiO_2_ slabs the presence of Ir near the TiO_2_ surface, which would strengthen the metal adhesion and lead to the experimentally observed higher stability against sintering of (bulk-immiscible) Au-Ir NPs as compared to Au NPs^33^. The high-resolution AC-STEM images of Fig. 3(b,d) show that the Rh_ball_Au_cup_ structure, which had never been reported for any bimetallic system to our knowledge, is quite complex and should be further investigated in the future. The transition from the low-temperature structure to this ball-cup one might result from a thermally-activated partial dewetting of the NPs from the support together with their tendency to enrich their surface with gold.

## Conclusions

Through the combination of model catalyst synthesis, AC-STEM-EDX characterization and DFT simulations, original nanostructures could be evidenced and investigated for the Au-Rh system supported on rutile titania. After mild annealing in hydrogen (350 °C) corresponding to typical catalyst activation and reaction conditions, a Janus structure with Rh in contact with the support is formed. This stacked chemical configuration (Au/Rh/TiO_2_) is driven by the Au-Rh demixing tendency, the lower surface energy of Au, and the preferential affinity of Rh with the substrate. After severe annealing (700 °C), a Rh_ball_Au_cup_ configuration, intermediate between Rh@Au core-shell and Janus, is preferred. These two types of structures are expected for other oxide-supported bimetallic systems and may lead to cooperative catalytic effects, such as those observed for AuRh/TiO_2_ in hydroprocessing reactions.

## Methods

### Experimental methods

TiO_2_ rutile nanorods were prepared using a simplified procedure based on a hydrothermal method reported by Li and Afanasiev^34^. 10 g of commercial Degussa P25 TiO_2_ (50 m^2^/g) and 100 mL of 15 wt% H_2_SO_4_ solution were mixed in a Teflon reactor and placed in a sealed autoclave kept at 200 °C for 15 days. The obtained solid was washed several times with 0.1 M NH_4_NO_3_ to remove adsorbed sulphate, then washed with distilled water, dried at 100 °C overnight, and calcined at 350 °C in air for 2 h.

Au-Rh NPs were prepared by a colloidal chemical (co)reduction route adapted from Toshima, Prati, Hutchings, and co-workers^12^,^35^,^36^,^37^,^38^. The metal precursors were HAuCl_4_.3H_2_O (Strem Chemicals, 99.9%, 49 wt% Au) and RhCl_3_.*n*H_2_O (Sigma-Aldrich, 99.9%, 38–40 wt% Rh). In a first step, a 200 mL aqueous solution containing the two metallic precursors was prepared by adding the amounts of precursors necessary for reaching a total metal loading of 3 wt%, with 50:50 at% Au:Rh composition. Next, a 1 wt% aqueous solution of a stabilizing agent, polyvinyl alcohol (PVA, Mw = 10,000) was added to the preceding solution while keeping always a mass ratio m_PVA_/m_Au+Rh_ of 1.2. A solution of 0.1 M NaBH_4_, freshly prepared and kept at 0 °C before use, was then dropped under stirring to the metallic precursors solution with a molar ratio n_NaBH4_/n_Au+Rh_ of 5. Stirring was then maintained for 30 min to allow the complete decomposition of the remaining NaBH_4_ excess. The solution was then acidified to pH 3.5 by addition of HCl 0.01 M in order to favour the sol immobilization onto the TiO_2_ support. The amount of support necessary for reaching the final metal loading was then added and stirring was kept for 3 h. Finally, the material was filterted, washed with hot distilled water (70 °C) several times, and dried at 100 °C overnight. An *ex situ* treatment consisting in heating the samples to 350 °C or 700 °C (10 °C/min, 3 h plateau) in hydrogen flow (5 mL/min, 1 atm) was applied using a dedicated bench.

The Au and Rh loadings of the Au-Rh/TiO_2_ catalysts were determined by inductively coupled plasma optical emission spectroscopy (ICP-OES) using an Activa spectrometer from Horiba Jobin Yvon. In order to dissolve them completely, the samples were treated with a mixture of H_2_SO_4_, *aqua regia* and HF at 250–300 °C. The measured metal loadings were 1.5–2.0 wt% for Au (target 2.0 wt%), and 0.6–0.7 wt% for Rh (target 1.0 wt%). As a result, the overall Au:Rh composition was comprised between 63:37 at% (samples 1 and 2; sample 2 is the supported counterpart of sample 1’s unsupported colloids) and 55:45 at% (sample 3). The unsupported and supported Au-Rh NPs are denoted AuRh@PVA and AuRh/TiO_2_, respectively, in the main text.

Scanning transmission electron microscopy (STEM) observations were conducted using a JEM-2100F (FEG, 200 kV) equipped with a probe aberration corrector, a high-angle annular dark field (HAADF) detector, and an energy dispersive X-ray (EDX) spectrometer. For STEM investigations, the dry samples were crushed in air using glass slides and were casted on copper TEM grids covered with holey carbon (supported nanoparticles) or lacey carbon (unsupported nanoparticles).

### Theoretical methods

The calculations were performed using density functional theory (DFT) as implemented in the VASP code^27^. The generalized gradient approximation (GGA) was employed within the Perdew-Burke-Ernzerhof (PBE) parameterization for the exchange-correlation energy functional^28^. All the calculations were spin-polarized, with valence electrons treated explicitly, while the ionic cores were represented by the projected augmented wave (PAW) method^29^,^30^. To avoid spurious periodic interactions, unsupported clusters were placed into a sufficiently large supercell that ensures ~10 Å separation by vacuum. For the same reason, the supported cluster studies were carried out on a 3 × 3 TiO_2_(110) surface using a slab of 9 atomic layers. Although the lateral cluster separations are ~7 Å and ~9 Å on the 3 × 3 TiO_2_(110) surface, the total energy change was found to be less than 0.02 eV for both Au_79_ and Rh_79_ clusters. Therefore, there was no need to go to the larger surface cell size of 4 × 4 TiO_2_(110), which is computationally much more expensive. The Γ point was used to sample the Brillouin zone. All atoms except those of the bottom three atomic layers of the TiO_2_(110) slab were relaxed until the forces on the atoms became lower than 0.01 eV/Å, and the electronic ground states were determined by requiring a total energy convergence of 10^−6^ eV. For the stability comparisons, a mixing energy term was calculated^31^:


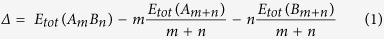


in which the total energy (*E*_*tot*_) of the nanoalloy *A*_*m*_*B*_*n*_ is compared to those of the pure metal (*A* or *B*) clusters of the same size (*m *+ *n*). Hence, a negative value of *Δ* means an energy decrease upon mixing, and therefore a more stable cluster. To determine the support effect on nanoalloy energetics, *Δ*′ was defined in the same manner as Δ by replacing the *E*_*tot*_ values with the supported-cluster counterparts (*E*_*tot*_′). The adsorption energies were calculated using the total energy differences of the separated and combined cluster and support systems:


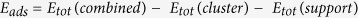


## Additional Information

**How to cite this article**: Piccolo, L. *et al*. Understanding and controlling the structure and segregation behaviour of AuRh nanocatalysts. *Sci. Rep.* **6**, 35226; doi: 10.1038/srep35226 (2016).

## Supplementary Material

Supplementary Information

## Figures and Tables

**Table 1:** Results of DFT calculations.

Composition	Structure	Δ (eV)^a^	E_tot_ (eV)^b^	Contact to TiO_2_	Δ′ (eV)^a^	E_tot_′ (eV)^b^	E_ads_ (eV)^c^
Au_60_Rh_19_	Rh_core_Au_shell_	−5.25	−290.58	Through Au	−3.83	−3119.42	−3.39
Au_50_Rh_29_	Rh_core_Au_shell_	−4.48	−324.53	Through Au	−2.15	−3153.38	−3.45
	Janus	2.67	−317.38	Through Au	5.96	−3145.47	−2.64
				Through Rh	−1.51	−3152.79	**−9.96**
		3.49	−316.56	Through both	4.45	−3146.84	−4.83
	Rh_ball_Au_cup_	0.33	−319.72	Through Au	3.36	−3147.92	−2.76
				Through Rh	**−3.31**	**−3154.59**	−9.43
				Through both	−0.96	−3151.31	−6.14
Au_45_Rh_34_	Janus	3.56	−333.85	Through Au	7.50	−3161.63	−2.32
				Through Rh	0.12	**−3169.01**	**−9.71**
Au_41_Rh_38_	Rh_ball_Au_cup_	−1.69	−352.99	Through Au	0.36	−3181.67	−3.23
				Through Rh	**−2.82**	**−3184.85**	**−6.41**
				Through both	−0.25	−3183.65	−5.21
Au_19_Rh_60_	Au_core_Rh_shell_	26.02	−401.67	Through Rh	23.18	−3238.74	−11.62

^a^Mixing energies for free (Δ) and supported (Δ′) clusters; ^b^Total energies for free (E_tot_) and supported (E_tot_′) clusters; ^c^Adsorption energies of supported clusters (E_ads_). The lowest (negative) energy values for each stoichiometry are bolded.
