# Supplementary material for: Understanding and controlling the structure and segregation behaviour of AuRh nanocatalysts
Source: Sci Rep. 2016 Oct 14;6:35226. doi: 10.1038/srep35226 (PMC5064371; doi:10.1038/srep35226)
Supplement: Supplementary Information [file srep35226-s1.pdf]

Supplementary Information

Understanding and controlling the structure and segregation behaviour of AuRh nanocatalysts

Laurent Piccolo,[a,*] Z.Y. Li,[b,*] Ilker Demiroglu,[c] Florian Moyon,[d] Zere Konuspayeva,[a] Gilles Berhault,[a] Pavel Afanasiev,[a] Williams Lefebvre,[d] Jun Yuan,[e] Roy L. Johnston[c]

[a] *Institut de recherches sur la catalyse et l'environnement de Lyon (IRCELYON), UMR 5256 CNRS & Université Claude Bernard - Lyon 1, 2 avenue Albert Einstein, F-69626 Villeurbanne, France.*

[b] *Nanoscale Physics Research Laboratory, School of Physics and Astronomy, University of Birmingham, Birmingham B15 2TT, United Kingdom.*

[c] *School of Chemistry, University of Birmingham, Birmingham B15 2TT, United Kingdom.*

[d] *Groupe de physique des matériaux, UMR 6634 CNRS & Université de Rouen, Avenue de l'Université, CS 70012, 76801 Saint-Etienne-du-Rouvray Cedex, France.*

[e] *Department of Physics, University of York, York, YO10 5DD, United Kingdom.*

* Corresponding authors. E-mails: laurent.piccolo@ircelyon.univ-lyon1.fr; z.li@bham.ac.uk

Additional HAADF-STEM images of supported AuRh nanoparticles heated to 350 °C

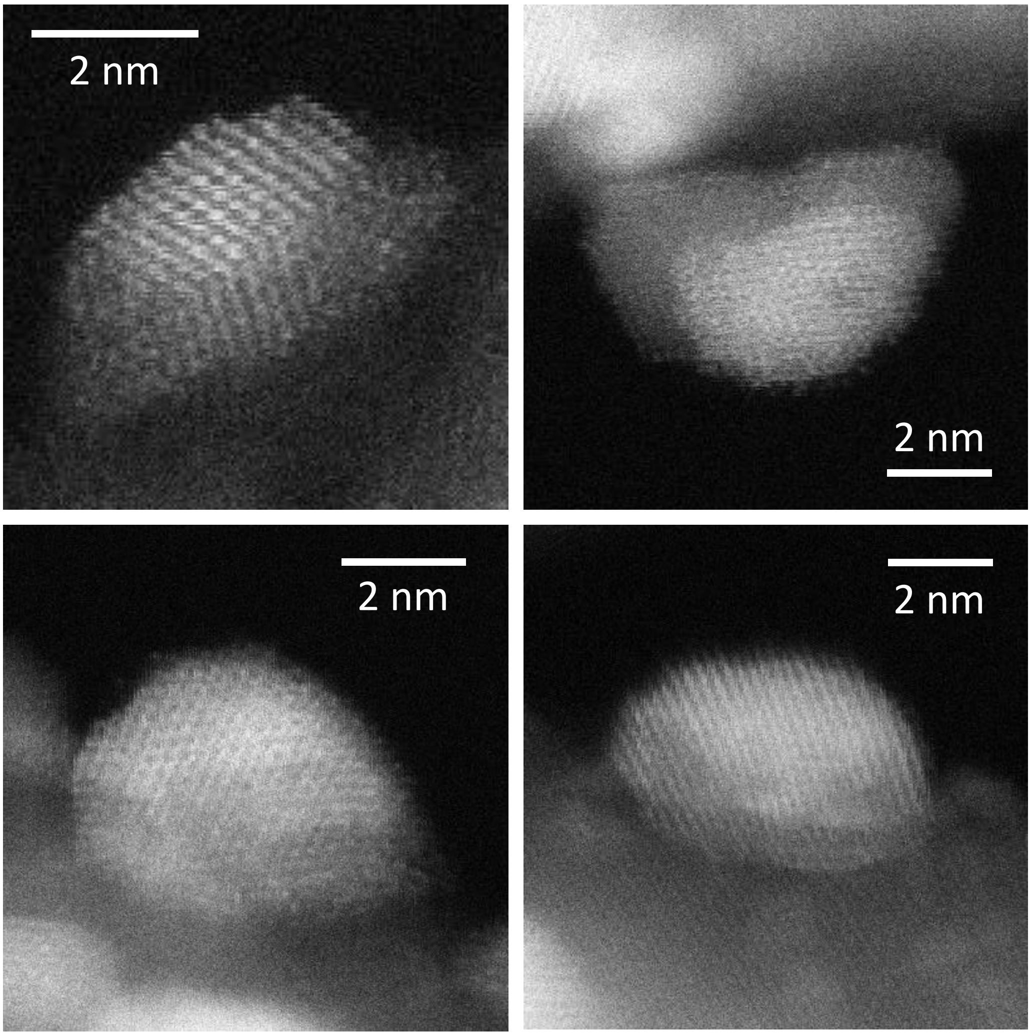

Figure S1. STEM-HAADF images of AuRh/TiO_2 (sample 2, $Au_{63}Rh_{37}$ average composition) pretreated in H_2 at 350 °C.

Reconstruction of a nanoparticle from the quantification of HAADF-STEM image intensities

The way a 3D model of an Au-Rh particle has been built from the image of Figure 2d is detailed hereafter. The method is based on the quantification of HAADF-STEM intensities. The methodology used for atom counting was developed by Van Aert *et al.*[1–3] For a thin specimen, the HAADF-STEM images intensities $f_{k,l}(\vec{r}_{k,l};\theta)$ can be expressed as the convolution of an object function $O(\vec{r}_{k,l};\theta)$, which depends on a set of unknown structure parameters θ, and the point spread function (PSF) of the electron probe $P(\vec{r}_{k,l})$, which depends on the probe parameters:

$$f_{k,l}(\vec{r}_{k,l};\theta) = O(\vec{r}_{k,l};\theta) * P(\vec{r}_{k,l}) \qquad (1)$$

where $\vec{r}_{k,l}$ is the vector pointing to pixel (k,l). The integrated intensity, represented by the object function $O(\vec{r}_{k,l};\theta)$, should be described as a superimposition of Gaussian functions:

$$O(\vec{r}_{k,l};\theta) = \chi + \sum_{m=1}^{M} \alpha_m e^{-\frac{(x-x_m)^2+(y-y_m)^2}{2\rho^2}} \qquad (2)$$

where χ is a constant background, ρ the width of a Gaussian peak, α_m the weight of a Gaussian peak, x_m and y_m the x- and y- coordinates of the $m_i th$ atomic column, and M the number of atomic columns. Then, the histogram of the integrated intensities can be fitted with a sum of Gaussian functions:

$$p(V_i|\Psi_G) = \sum_{g=1}^{G} \pi_g \frac{1}{\sqrt{2\pi\sigma}} e^{-\frac{(V_i-\mu_g)^2}{2\rho^2}} \qquad (3)$$

where $p(V_i|\Psi_G)$ is the probability that the intensity V_i would be estimated for a particular atomic column i, $\Psi_G = (\pi_1, \dots, \pi_G, \mu_1, \dots, \mu_G, \rho)$ is a vector containing the unknown parameters of the probability distribution, μ_g corresponds to the position of the mean intensity value of a column composed of g atoms, G is the number of Gaussian functions, and σ corresponds to the fluctuation of intensities. To adjust the histogram of the integrated intensities, determining G is crucial. Van Aert *et al.*[1–3] have demonstrated that this parameter can be deduced from the drop of the integrated classification likelihood criterion (ICL).

The robustness of this methods has been tested on high-resolution HAADF-STEM image simulations and it has been demonstrated that coupling the deconvolution by the PSF and the ICL calculation provides a very reliable atom counting methodology.[4]

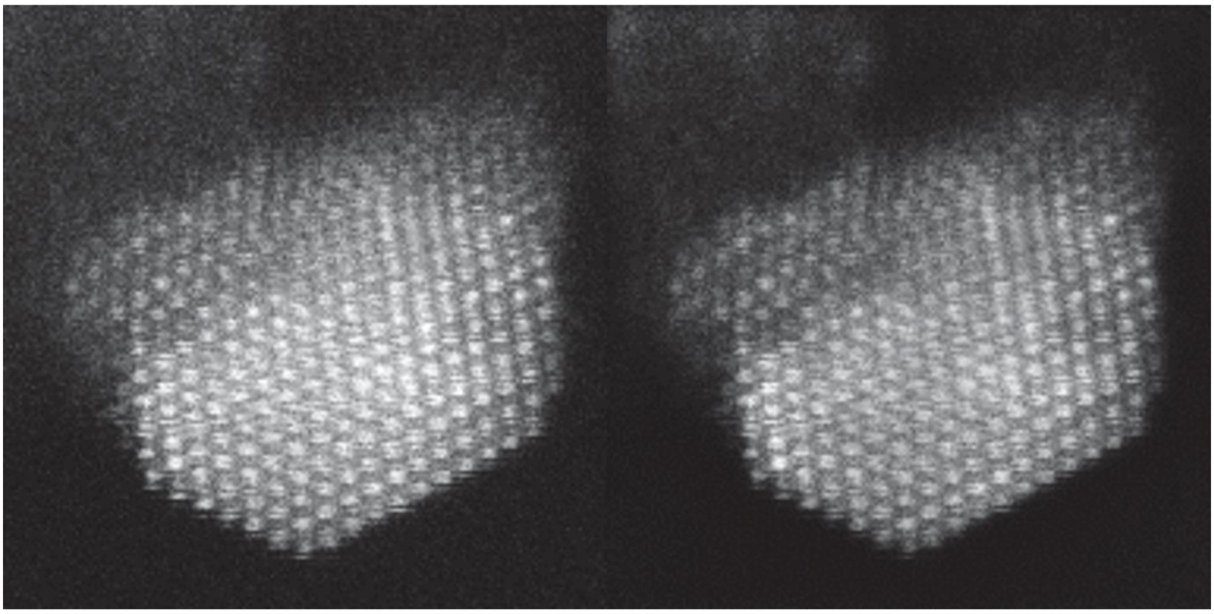

Figure S2. *Left*: original image (identical to that in Figure 1d). *Right*: Image devonvoluted by the PSF of the electron probe.

In Figure S1, the deconvolution by the PSF was performed by using the MatLab *deconvlucy* function with 3 iterations. The number of iterations was determined by choosing the best defined ICL for further fitting of the intensity histograms. In order to build a 3D model of the particle using a single projection, some hypotheses must be formulated. It was assumed that the columns are either pure Au or pure Rh, and that the interface between Au and Rh regions in the particle is perpendicular to the image. These hypotheses are supported by the observations made for other particles (Figure 2). The differentiation between the Au columns and the Rh columns was performed from the intensities and the crystal lattice variation. Then, two atom count maps were calculated, one for the Rh part and the other for the Au part. These two assumptions can lead to some misrepresentation at the interface but are necessary to build a model from a single projection. The results are shown in Figure S2. The reliability of the atom counting method is directly connected to the fit of the histogram with Gaussian functions. Indeed, as it can be observed in Figures S2d and S2h, the Gaussians of the nearest components can overlap. Consequently, if the integrated intensity of one particular column is located in the overlapping of two Gaussians, the number of atoms in this column can be misevaluated by one atom.

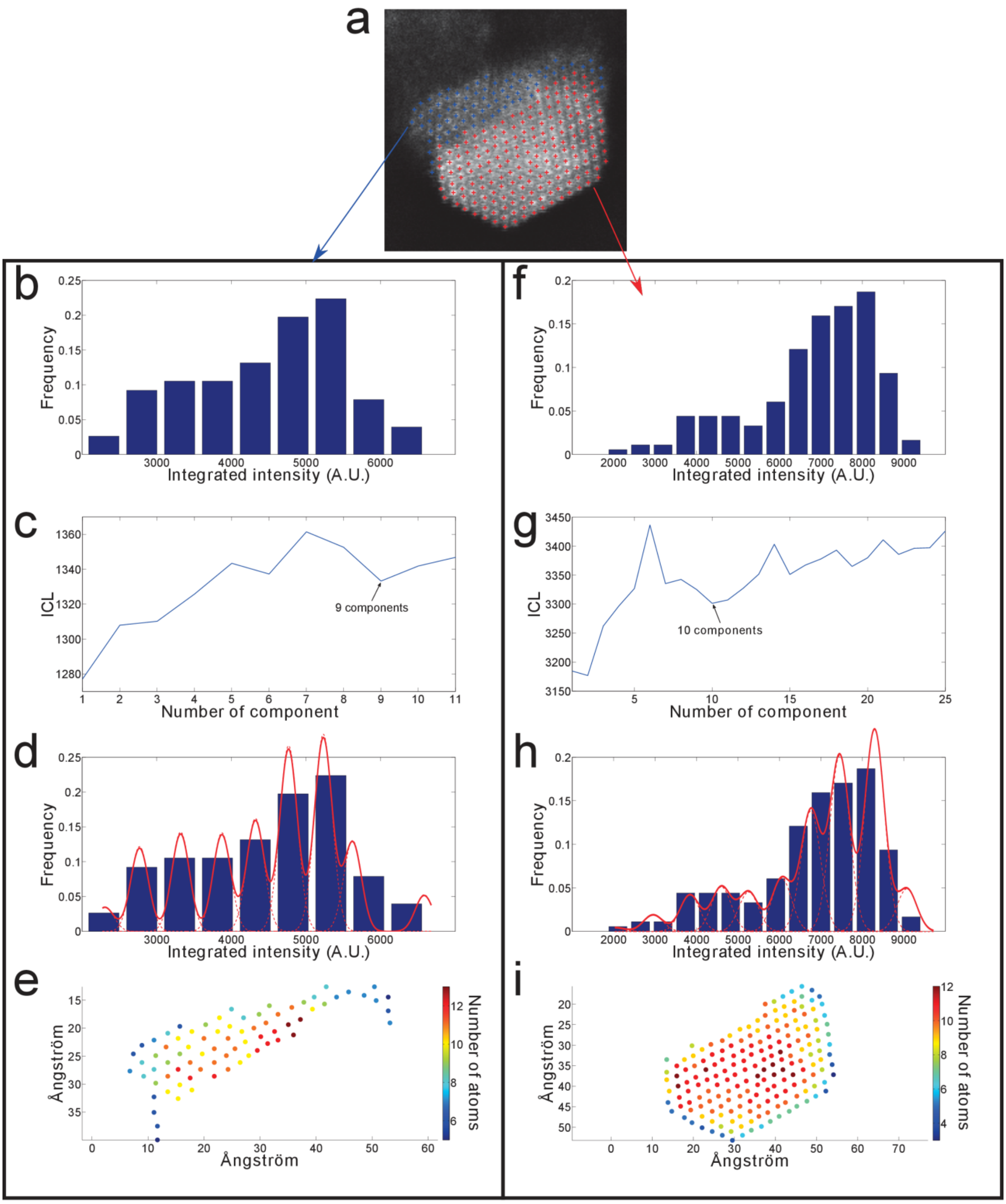

Figure S3. (a) STEM-HAADF image of the particle with red and blue crosses corresponding to the atomic columns selected as Au and Rh ones, respectively. (b-e) Atom counting for the Rh part: (b) represents the histogram of the atomic column's integrated intensities, (c) the ICL function of the number of components (the ICL drop is observed for 9 components), (d) the histogram in (b) fitted with 9 Gaussian functions, and (e) the atom count map. (f-i) Similar analysis for the Au part.

After the determination of the number of atoms in each atomic column, a simple back-projection on a FCC lattice was carried out (Figure S3). We supposed a symmetrical particle along the projection axis *i.e.* along the electron beam propagation. Although Van Aert *et al*. have shown the ability to reconstruct exactly in three dimensions one particle with two different HAADF-STEM image orientations,[1,2,5–7] with only one orientation this assumption (symmetrical particle) is necessary. Moreover, as shown in Figures 2f and S3b, this hypothesis appears reasonable since we are able to visualize the facets of a truncated octahedron, as expected for a gold nanoparticle.[8]

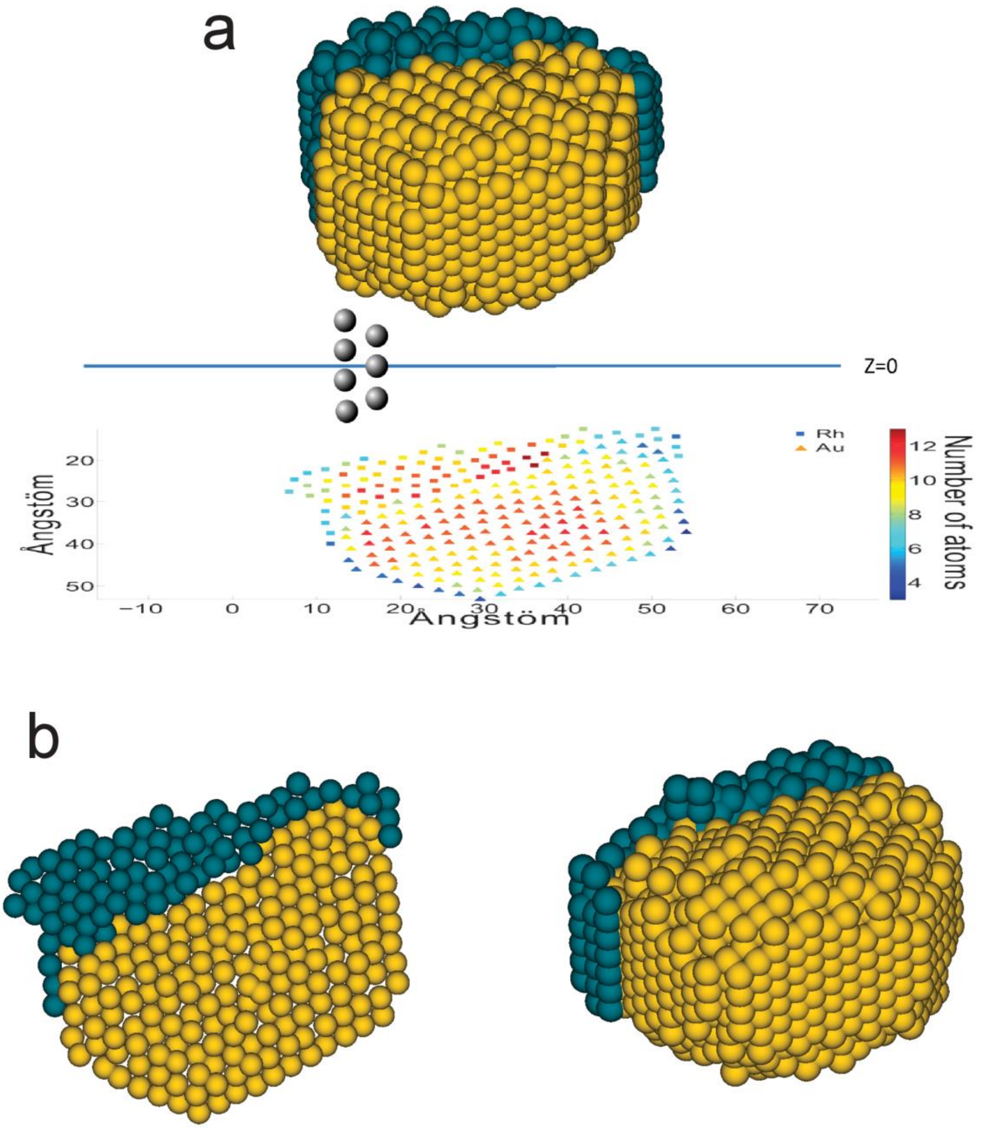

Figure S4. (a) Atom count map for Rh and Au, and representation of the back-projection with the assumption of symmetrical particle along *z* axis, *i.e.* along electron beam propagation. (b) Visualization of the particle model for an orientation similar to that of the image in Figure 2d (*left*) and for a random orientation (*right*).

STEM-EDX analysis of supported AuRh nanoparticles heated to 700 °C

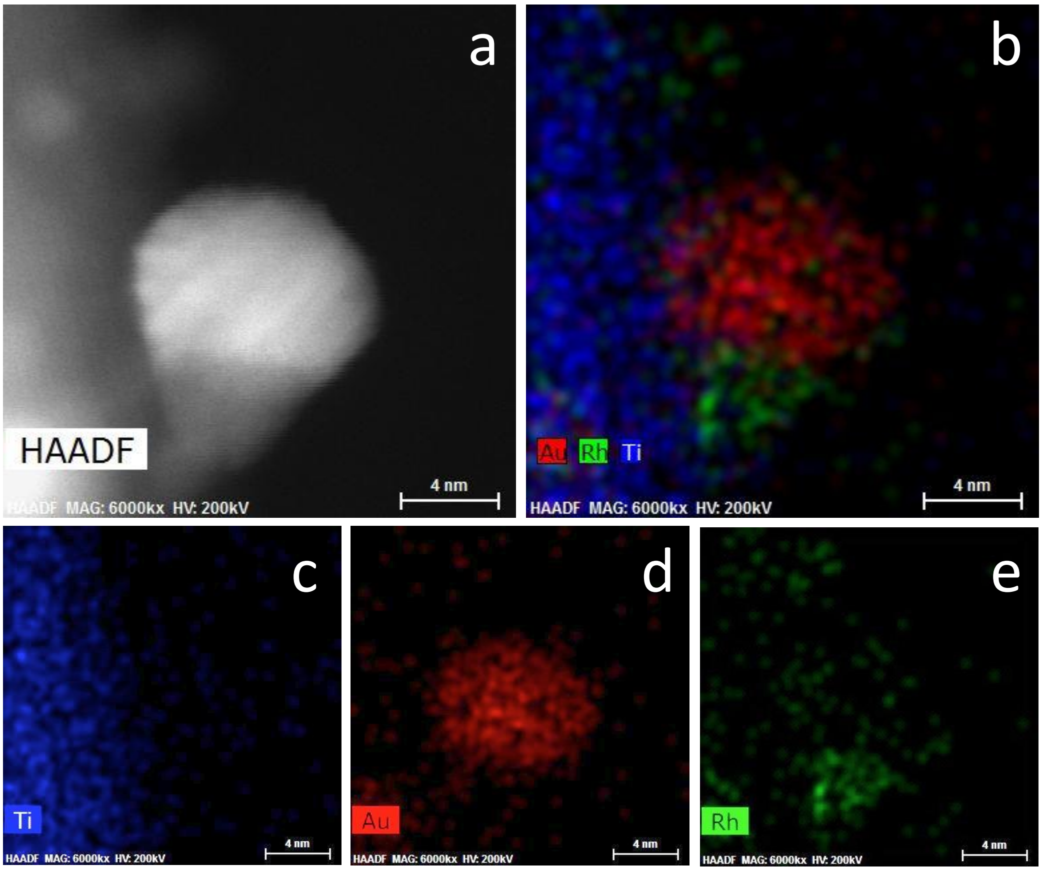

Figure S5. a) EDX-HAADF image of the particle shown in Figure 3(a,b). b) Combined EDX map of the same region. c-e) Individual elemental EDX maps. Size of the scale bars: 4 nm.

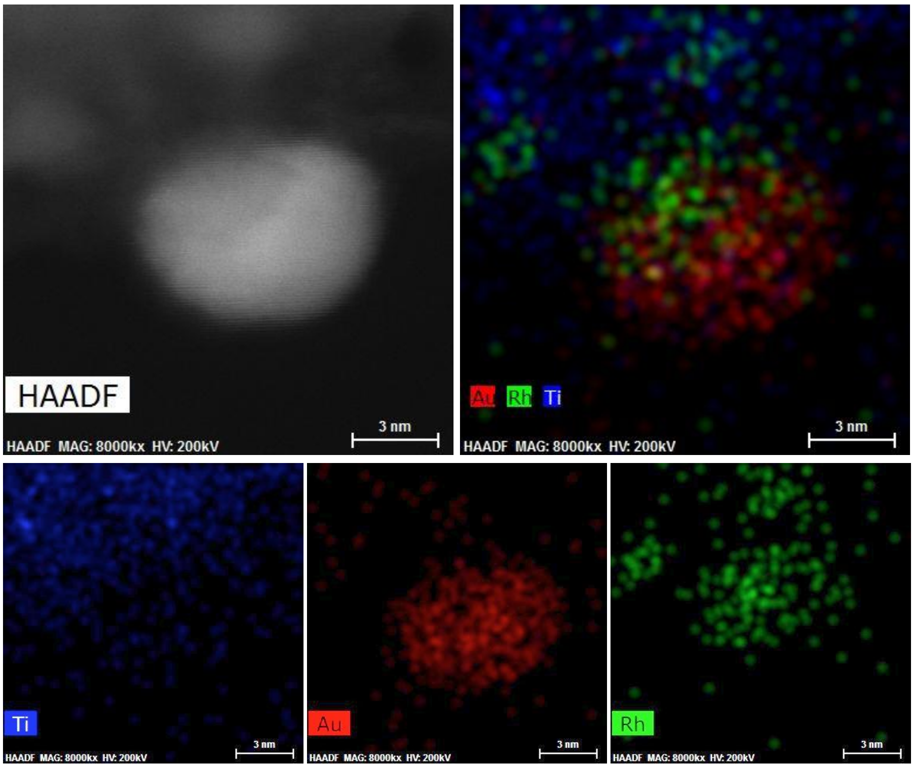

Figure S6. EDX-HAADF image and elemental maps of a ball-cup particle (sample 3). Size of the scale bars: 3 nm.

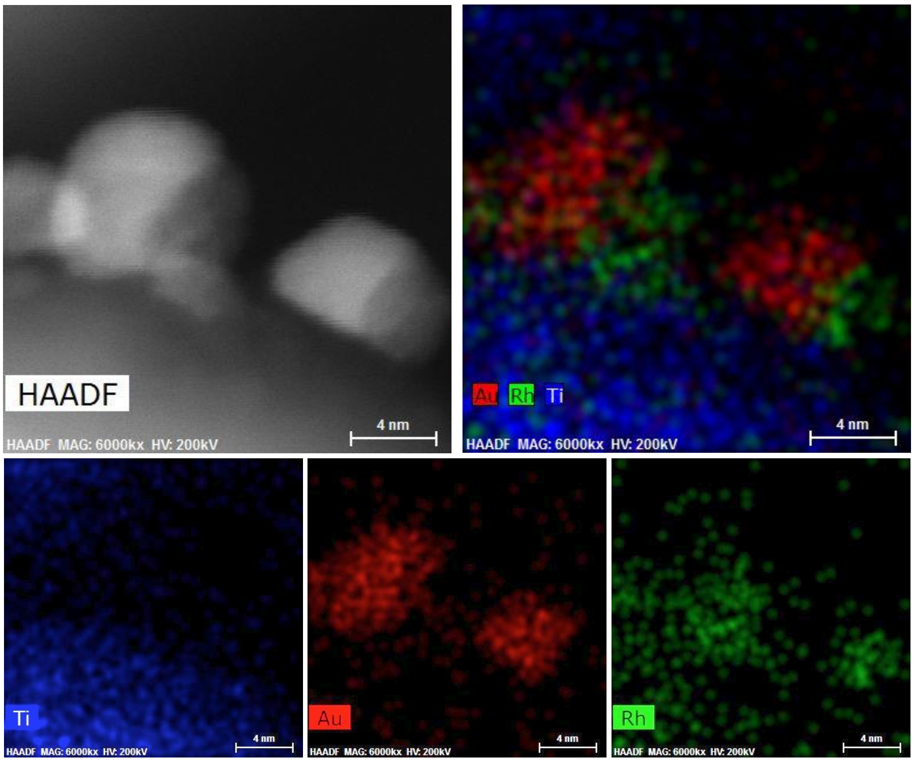

Figure S7. EDX-HAADF image and elemental maps of two ball-cup particles (sample 3). Size of the scale bars: 4 nm.

References

1. De Backer, A., Martinez, G. T., Rosenauer, A. & Van Aert, S. Atom counting in HAADF STEM using a statistical model-based approach: Methodology, possibilities, and inherent limitations. *Ultramicroscopy* **134,** 23–33 (2013).
2. Van Aert, S. *et al.* Procedure to count atoms with trustworthy single-atom sensitivity. *Phys. Rev. B* **87,** (2013).
3. Martinez, G. T., Rosenauer, A., De Backer, A., Verbeeck, J. & Van Aert, S. Quantitative composition determination at the atomic level using model-based high-angle annular dark field scanning transmission electron microscopy. *Ultramicroscopy* **137,** 12–19 (2014).
4. Lefebvre, W. *et al.* HAADF-STEM atom counting in atom probe tomography specimens: Towards quantitative correlative microscopy. *Ultramicroscopy* (2015). doi:10.1016/j.ultramic.2015.02.011
5. Bals, S. *et al.* Three-Dimensional Atomic Imaging of Colloidal Core–Shell Nanocrystals. *Nano Lett.* **11,** 3420–3424 (2011).
6. Van Aert, S., Batenburg, K. J., Rossell, M. D., Erni, R. & Van Tendeloo, G. Three-dimensional atomic imaging of crystalline nanoparticles. *Nature* **470,** 374–377 (2011).
7. Jones, L., MacArthur, K. E., Fauske, V. T., van Helvoort, A. T. J. & Nellist, P. D. Rapid Estimation of Catalyst Nanoparticle Morphology and Atomic-Coordination by High-Resolution Z-Contrast Electron Microscopy. *Nano Lett.* **14,** 6336–6341 (2014).
8. Baletto, F., Ferrando, R., Fortunelli, A., Montalenti, F. & Mottet, C. Crossover among structural motifs in transition and noble-metal clusters. *J. Chem. Phys.* **116,** 3856 (2002).
